# The Long Journey of a Bird: From Sangay to the Laboratory

**DOI:** 10.4269/ajtmh.26-0073

**Published:** 2026-08-11

**Authors:** Glenda Pozo-Zamora, Emily Cadena-Castro, Ángel Sebastián Rodríguez-Pazmiño

**Affiliations:** ^1^ Instituto Nacional de Biodiversidad (INABIO), Quito, Ecuador;; ^2^ One Health Research Group, Universidad de Las Américas, Quito, Ecuador

Before leaving the camp, we left a large bag of food behind, intending to return to that spot and conduct our research there for about 5 days. My colleagues wanted to leave the food inside the small house. I was wary of leaving it there because it was not secure. So, I said to them, “How about we hide or bury the bag to prevent anyone from taking it?”

My companions responded, teasing me a little: “No need because no one will come this far; they won’t take the food. Relax, Glenda.”

I reluctantly agreed.

It was 2015. I had joined an expedition to Sangay National Park in southern Ecuador, a remote region where we set out to document birds and small mammals inhabiting the eastern slopes of an area called Guabisai. At the time, our goal was taxonomic and ecological: to understand what species lived there. We did not yet know that some of the specimens that we collected would, years later, help us search for avian pathogens and contribute to questions about ecosystem health and disease emergence.

There were four of us there, and the park rangers guided us through steep pastures and narrow trails accompanied by mules carrying our equipment: bird nets, mammal traps, work materials, food, and the essentials for several days in the field. On a difficult stretch, one of the mules turned around, and we almost lost part of the load. Even so, we managed to reach our first objective, Camp 1, where there was nothing more than a small 2 × 2-meter wooden hut where we could leave our gear. Outside, we set up a small tent and slept there that night. The park rangers accompanied us to that point and then, returned with the mules.

The hike on the second day was not as steep, but it was much muddier. It took us several hours to make progress, carrying the traps, some of the food, and our personal belongings ourselves. When we finally reached the sampling site, we organized ourselves to set up traps, observe, and capture animals.

We set up mist nets in forest clearings and along natural flight paths where birds travel at dawn and dusk. We also set traps for small mammals near low vegetation. The site was pristine; each capture represented a piece of information about a little-studied ecosystem.

Amid constant humidity, exhaustion, and difficult terrain, we checked nets and traps again and again. But, the effort was worth it. We collected around 20 bird specimens and some small mammal species.

After 5 intense days, we returned to Camp 1 to restock food and work for another 5 or 6 days. There, we discovered that the bag had disappeared, just as I had feared. We only had a can of sardines, a little coffee, and some popcorn left. We had no signal to call for help, and the situation quickly became distressing. The park rangers were supposed to return after 6 days, and we did not know how we would manage until then.

One of my companions had a Nokia 1100, one of those old phones that were real workhorses. He climbed a tree in search of a signal, and against all odds, he managed to contact the park rangers and ask them to return as soon as possible.

That night, we slept as best we could. The next day, we began the return journey on our own, hoping to meet them somewhere along the way. We walked for almost an entire day: exhausted, hungry, and on the verge of fainting. We pushed ourselves physically and mentally like never before.

When we finally reunited with the park rangers, they welcomed us with chaulafán—fried rice with meat, vegetables, and soy sauce. It was without a doubt the best chaulafán of my life.

Today, the 20 bird species that we collected from Sangay National Park have moved from forest trails and hunger to museum drawers and laboratory benches. In the Instituto Nacional de Biodiversidad (INABIO), the specimens have been collected, labeled, preserved, and cared for over the years. Now, to revisit past ecosystems from the perspective of infectious disease, I have entrusted these birds and other specimens to Universidad de Las Américas (UDLA) through its student, Emily.

Trying to identify the different birds, Emily once said to me while she was doing an internship at INABIO: “But they look the same.” So, I encouraged her to look more closely: the shape of the beak, the patterns on the plumage, and the subtle differences that are easy to miss at first glance. We would open books on Ecuadorian birds together and compare them again and again until, little by little, she began to notice what she had not seen before.

Through this collaboration with UDLA, nearly 400 tissue samples collected over many years of expeditions, including those from Sangay, have entered a new phase of study. Under Sebastián’s guidance, a PhD student at UDLA, Emily is now training in molecular techniques that allow us to detect pathogens’ genetic material in those preserved tissues. Each result raises new questions. Are these pathogens expected in migratory species? Do they reflect environmental change? Could they signal broader patterns in wildlife health?

When I think about the future of bird and pathogen research in Ecuador, I have mixed feelings. It gives me hope to see how bird tourism, birding, and birdwatchers’ curiosity have brought many people closer to science. But, I am concerned about the lack of funding and specialized personnel and the growing difficulties in conducting expeditions because of the country’s terrible insecurity. But now, those 20 bird species collected in Sangay National Park 11 years ago are part of a new chapter: the search for avian pathogens. What began as a difficult journey through the forest now continues in drawers, tubes, and data, opening the door to new expeditions, questions, and collaborations.

